# Areca nut chewing and dependency syndrome: Is the dependence comparable to smoking? a cross sectional study

**DOI:** 10.1186/1747-597X-6-23

**Published:** 2011-08-18

**Authors:** Saira S Mirza, Kashif Shafique, Priya Vart, Moin I Arain

**Affiliations:** 1 Department of Physiology, Institute of Basic Medical Sciences, Dow University of Health Sciences, Karachi, 74000, Pakistan; 2 Centre of Population & Health Sciences, Department of Public Health and Health Policy, University of Glasgow, Glasgow, G12 8RZ, UK; 3 Department of Medicine, Isra University, Hyderabad, 71000, Pakistan

**Keywords:** Dependency syndrome, Areca nut, tobacco

## Abstract

**Background:**

Areca nut is the seed of fruit oriental palm known as *Areca catechu*. Many adverse effects of nut chewing have been well documented in the medical literature. As these nuts are mixed with some other substances like tobacco and flavouring agents, it has been hypothesized that it might also cause some dependency symptoms among its users. Therefore, the objective of this study was to investigate dependency syndrome among areca nut users with and without tobacco additives and compare it with dependency associated with cigarette smoking among the male Pakistani population.

**Methods:**

This was an observational cross sectional study carried out on healthy individuals, who were users of any one of the three products (areca nut only, areca nut with tobacco additives, cigarette smokers). Participants were selected by convenience sampling of people coming to hospital to seek a free oral check up. Information was collected about the socio-demographic profile, pattern of use and symptoms of dependency using the DSM-IV criteria for substance dependence. We carried out multiple logistic regressions to investigate association between socio-demographic profile, pattern of substance use and dependency syndrome.

**Results:**

We carried out final analysis on 851 individuals, of which 36.8% (n = 314) were areca nut users, 28.4% (n = 242) were the chewers of areca with tobacco additives and 34.7% (n = 295) were regular cigarette smokers. Multivariate analyses showed that individuals using areca nut with tobacco additives were significantly more likely to have dependency syndrome (OR = 2.17, 95% CI 1.39-3.40) while cigarette smokers were eight times more likely to have dependency syndrome as compared to areca nut only users.

**Conclusions:**

Areca nut use with and without tobacco additives was significantly associated with dependency syndrome. In comparison to exclusive areca nut users, the smokers were eight times more likely to develop dependence while areca nut users with tobacco additives were also significantly more likely to suffer from the dependence.

## Background

Areca nut is the seed of the fruit of the tropical palm tree, *Areca Catechu*. This tree bears fruit throughout the year and areca nut is obtained from it, which is a basic ingredient of widely used chewing products[1]. Thin slices of nuts either natural or processed are then mixed with a variety of substances including slaked lime (calcium hydroxide) and spices such as carda-mom, coconut, and saffron [2,3]. Most importantly these nuts are also mixed with some tobacco products.

The areca nut is considered to be the fourth most commonly used psychoactive substance after the tobacco, alcohol and caffeine and it has been suggested that approximately 10% of the World's population chew it regularly[4]. The usage of areca nut is indigenous to India, Sri Lanka, Maldives, Bangladesh, Myanmar, Taiwan and numerous islands in South Pacific. It is also consumed in parts of Thailand, Indonesia, Malaysia, Cambodia, Vietnam, Philippines, Laos, and China and in the migrant individuals from these countries [5,6]. Among the populations residing in South and East Asia, areca nut chewing is socially acceptable. The epidemiological data pertaining to areca nut use in Pakistan has been reported in different subgroups and rates vary depending on the sample studied and the geographical area in which the study was conducted [3,6,7]. However nut chewing is a common practice which usually starts in school life and remains prevalent till adulthood.

Many epidemiological studies have revealed that continuous use of areca nut is associated with many adverse health effects from oral leukoplakia to submucous fibrosis which is subsequently linked with cancer risk [4,5,8]. WHO International Agency for Research on Cancer Monograph Working Group (2009) highlighed that the evidence on areca nut and its assosciation with oral, pharyngeal and esophageal cancer is sufficient to establish a causal link[8].

Areca nut chewing has been claimed to manifest a sense of well-being, euphoria, salivation, warm sensation of the body, low to moderate sweating and palpitations[9]. These characteristics of areca nut along with its ingredients highlight the existence of some dependency symptoms among its users.

Small scale observational studies have reported the existence of dependency syndrome among the users of areca products [10,11]. A relatively larger study, which was conducted in India reported that areca nut use alone and more so with tobacco additives, is associated with the development of a dependency syndrome among significantly larger proportions of its users i.e. 39% and 79% respectively [9]. However little is confirmed about the dependency syndrome among areca nut users as compared to the symptoms of dependency among tobacco users (cigarette smokers).

Previous evidence highlights the need to investigate this condition among areca nut users, with and without tobacco additives, and tobacco users. Therefore, the present study was carried out to explore the dependency syndrome among the areca nut chewers in comparison with cigarette smokers. Furthermore, the study also has an objective to investigate the major determinants associated with the dependency syndrome among the areca nut chewers and cigarette smokers.

## Methods

We included healthy individuals from population for this study who were invited to outdoor patients department of Civil Hospital Karachi. The participants were provided a free oral check-up with an oral surgeon at this site. We only included those individuals in this study who were users of any one of the three products (i.e. Areca nut only or Areca nut with tobacco additives or just tobacco use in the form of cigarettes). We prepared a list of 50 areca nut products commonly available in market, so that individuals can be classified in groups accurately. This study was approved from the hospital authorities and an independent ethical committee reviewed and approved the study protocol.

### Inclusion criteria

All willing healthy male individuals between the ages of 16 to 35 who visited the outdoor patient department during January 2009 to December 2010 in response to the invitation were included in this study.

### Exclusion criteria

Individuals who refused to participate in the survey or were unable to understand Urdu (National language of Pakistan) were excluded from this survey. Individuals who visited the hospital due to current illness or follow up of a previous illness were also excluded.

### Instrument

Information was collected by using a pre-tested questionnaire, which contained the information about socio-demographic profile (including age, years of education, nature of job/study), pattern of substance use (frequency, number of years since use, daily consumption, type of substance and family history of use). A single page information sheet was designed to convey the aim and objectives of the study to the participants and verbal consent was obtained prior to employing the questionnaire.

Questions to determine the presence of substance dependence, we used The Diagnostic and Statistical Manual of Mental Disorders (DSM-IV) module for substance dependence. We translated the DSM-IV module of substance dependence to Urdu by using a committee approach. Six individuals participated in this committee who were bilingual and selected for their proficiency in both English and Urdu. Two of them translated the English questionnaire to Urdu at an initial stage, while another two judged the translation in relation to the original questionnaire. Then on second step the other two individual who had no prior knowledge about this DSM-IV module translated the Urdu version back to English. The two judges again compared the converted English version of questionnaire to original questionnaire and then all six members sat together to select the best translations which represents the original. This questionnaire along with the questionnaire related to demographics and substance abuse, were piloted to the first 25 participants of the study. Participants were asked about any difficulty in understanding and comprehension of questions. After assessing the validity, based on piloted questionnaire we then administered both questionnaires to the study participants. Questions were about the (1)tolerance (needing more to become intoxicated or discovering less effect with same amount) (2)withdrawal (characteristic withdrawal associated with this type of drug) (3)Using more or for longer periods than intended? (4**) **Desire to or unsuccessful efforts to cut down? (5) Considerable time spent in obtaining the substance or using, or recovering from its effects? (6) Important social, work, or recreational activities given up because of use? (7) Continued use despite knowledge of problems caused by or aggravated by use. Presence of any three of these during the same 12 months period confirmed the presence of dependency syndrome.

### Sample size estimation

We estimated the sample size to measure 10% difference of dependency prevalence (assuming 75% prevalence of dependency syndrome) between groups at 0.05 significance level using two sided comparison and power of 80%. Sample size was computed for both the *X^2 ^*using the Yates' continuity correction and Fisher Exact test. A sample of 630 participants was the minimum number required to be accrued to perform this survey.

### Data analysis

We used Stata software version 11 (StataCorp, College Station, TX, USA) to analyze the collected information. Participants were divided into three groups according to their substance consumption ("areca nut only", "areca with tobacco additives", "smokers only"). For comparison between variables we used, Analysis of Variance (ANOVA) with bonferroni adjustments for multiple comparisons of continuous and Kruskal Wallis Test for ordinal variables. We used multiple logistic regression using different explanatory variables to assess the association of dependency syndrome with different factors (i.e. socio-demographic and pattern of substance consumption).

## Results

### Basic characteristics of study sample

We collected the information from 900 individuals who were regular users of any of the three products (i.e. areca nut or areca with tobacco additives or cigarette smokers). Data from thirty five subjects lacked basic information about age, use of product and exact quantity of substance used, while another fourteen subjects missed some important questions related to dependency symptoms. Therefore, these forty nine individuals were excluded from the final analysis. Final analysis was carried out on 851 individuals, out of which 36.8% (n = 314) were areca nut users, 28.4% (n = 242) were the chewers of areca nut with tobacco additives and 34.7% (n = 295) were regular smokers. Basic analysis indicated approximately similar average age in all three groups (25.5 ± 5.53 vs 25.0 ± 5.62 vs 25.9 ± 5.81: p = 0.19). There was no significant difference of years of formal education between the three groups (11.3 ± 2.56 vs 11.8 ± 3.26 vs 11.6 ± 2.10). However significant differences were noted in the family history of substance use and the number of years since they began using these products (Table 1).

**Table 1:** Baseline characteristics of sample according to the type of substance used

	Type of substance use				
		
Characteristics	Number (n)	Areca nut only, %	Areca + tobacco, %	Smokers only, %	p value *
**Total participants (n)**	851	314 (36.8)	242 (28.4)	295 (34.7)	
**Age at screening (years)**					
Age 16-20	182	14	22.31	28.47	< 0.001
Age 21-25	311	42	32.64	33.9	
Age 26-30	181	28	16.12	18.31	
Age 31-35	177	15.9	28.93	19.32	
**Education (years)**					
< 10 years	112	7.96	16.94	15.59	< 0.001
10-12 years	490	57.96	39.67	71.86	
> 12 years	249	34.08	43.39	12.54	
**Family history of substance use**					
Absent	554	55.1	33.06	25.76	< 0.001
Present	297	44.9	66.94	74.24	
**Duration of use (years)**					
< 6 years	467	44.59	50	69.83	< 0.001
6-10 years	255	39.49	22.31	26.1	
> 10 years	129	15.92	27.69	4.07	

* p values were calculated using the chi square test.

### Dependency syndrome among users

The frequency of dependency syndrome between the three groups was significantly different. Among areca only users, 67.2% (n = 211) had dependency syndrome while 78.1% (n = 189) among areca with tobacco additives users and 89.8% (n = 265) of smokers had dependency syndrome.

When looking at the symptoms of dependence, we observed significant differences between different groups of substance users (table 2). Withdrawal symptoms, attempt to cut down and continue use were significantly higher among the cigarette smokers (p < 0.001), while excess use than intended and social or recreational activity given up were highest among the areca nut only users (p < 0.001). Excess use among areca users might be due to tastes of these nuts as different flavouring agents are added in it.

**Table 2:** Dependency symptoms among users of areca nuts, areca nut with tobacco additives and cigarette smokers

	Type of substance use						
**Symptom**	**Areca nut only**		**Areca + Tobacco**		**Smokers only**		**P-value**
	**N = 314**		**N = 242**		**N = 295**		
	**n**	**(%)**	**n**	**(%)**	**n**	**(%)**	

Tolerance	101	(32.2)	90	(37.2)	116	(39.3)	< 0.001
Withdrawal	87	(27.7)	158	(65.3)	204	(69.2)	< 0.001
Excess use	239	(76.1)	102	(42.2)	119	(40.3)	< 0.001
Attempt to cut down	93	(29.6)	152	(62.8)	218	(73.9)	< 0.001
Considerable time spent before reuse or recovery	88	(28.0)	100	(41.3)	135	(45.8)	< 0.001
Activity given up	175	(55.7)	95	(39.3)	65	(22.0)	< 0.001
Continue use despite known health hazards	195	(62.1)	138	(57.0)	256	(86.8)	< 0.001

p values calculated using the chi^2 ^test between the presence or absence of symptom and group of substances use

On univariate analysis, increasing age, family history of substance use and increasing duration of use were significantly associated with increased likelihood of dependency syndrome. Comparison between the groups while taking the areca only as a reference group showed that both areca nut with tobacco additives (OR = 1.74, 95% CI 1.18-2.56) users and smoking only group (OR = 4.31, 95% CI 2.76-6.73) were more likely to have dependency syndrome (Table 3).

**Table 3:** Univariate and multivariate analyses: factors associated with dependency syndrome

	Univariate analysis			Multivariate analysis*		
	
	Odds ratio (95% CI)			Odds ratio (95% CI)		
		p value	Wald chi^2^		p value	Wald chi^2^
**Age at screening**						
Age 16-20	1			1		
Age 21-25	1.65 (1.11-2.48)	0.014	2.45	1.76 (1.06-2.91)	0.03	2.21
Age 26-30	2.01 (1.25-3.23)	0.004	2.89	0.87 (0.45-1.69)	0.68	-0.41
Age 31-35	6.52 (3.43-12.39)	< 0.001	5.72	1.16 (0.37-3.64)	0.79	0.26
**Years of education**						
< 10 years	1			1		
10-12 years	1.11(0.68-1.81)	0.68	0.41	0.91 (0.52-1.60)	0.75	-0.32
> 12 years	1.07 (0.63-1.81)	0.81	0.24	0.91 (0.50-1.67)	0.76	-0.30
**Family history of substance use**						
Absent	1			1		
Present	2.29 (1.57-3.37)	< 0.001	4.26	2.36 (1.39-4.01)	0.002	3.16
**Duration of use (years)**						
< 6 years	1			1		
6-10 years	2.87 (1.90-4.33)	< 0.001	5.02	3.62 (2.16-6.07)	< 0.001	4.89
> 10 years	5.88 (2.91-11.91)	< 0.001	4.92	7.18 (2.08-24.82)	0.002	3.12
**Group**						
Areca only	1			1		
Areca with tobacco additives	1.74 (1.18-2.56)	0.005	2.82	2.17 (1.39-3.40)	0.001	3.4
Smokers only	4.31 (2.76-6.73)	< 0.001	6.44	7.87 (4.70-13.15)	< 0.001	7.87

* Multivariate model included all the co-variates listed in the table

In order to predict the likelihood of existence of dependency syndrome among the three groups of substance users a multiple logistic regression was used. We used the existence of dependency syndrome (on the basis of DSM-IV criteria) as a dependent variables while age, years of education, number of years since using, family history of use and type of substance used as explanatory variables (independent). Multivariate analysis also showed similar findings as univariate analysis except that age was no longer associated with dependency syndrome after adjustment for the duration of use (Table 3). Areca users with tobacco additives were more likely to have dependency syndrome (OR = 2.17, 95% CI 1.39-3.40) so were the smokers only group (OR = 7.87, 95% CI 4.70-13.15) as compared to areca only users after adjustments for age, education, family history of substance use and duration of use (Table 3). We did not find any significant association between years of full time education and dependency syndrome.

## Discussion

In this study we observed a trend of dependency syndrome between three groups. Nine out of 10 cigarette smokers had dependency syndrome, while 8 out of 10 areca users with tobacco additives had dependency syndrome. As anticipated the frequency of dependency syndrome among the areca users with tobacco additives and cigarette smokers was very high in this sample. This can be explained by the addictive potential of nicotine. We observed 78.1% existence of dependency syndrome among areca users with tobacco additives which is fairly consistent with a previous study published on Indian population[9]. Furthermore, we also observed a significant difference in existence of dependency syndrome between areca users with tobacco additives and cigarette smokers. However it is difficult to exactly differentiate and estimate the dependency symptom associated with areca nut or tobacco independently. Dependency syndrome among the areca nut only group was 67%, although high, but have been found slightly inconsistent in earlier studies as well [9,11] Both of these studies had smaller sample sizes, so the differences between studies and in our findings could be simply because of chance. Another possible explanation is that both studies had participants from both sexes while we only included male participants, so observed higher difference in our study could be because of inclusion of only male individuals. Previous studies were conducted in England and India, types of areca nut available in different regions of the World might explain some of the observed differences. Biological mechanism underlying the association of areca nut chewing with dependency syndrome is less clear, however Arecoline (a psychoactive agent), which is the main alkaloid of areca nut, has been thoroughly investigated and areca nut chewing has been suggested to produce a sense of well being, euphoria, heightened alertness, sweating, salivation and a hot body sensation [11]. Combination of these feelings could actually lead to tolerance from these products and that could ultimately result in the development of dependency syndrome.

Univariate and multivariate analysis illustrates the level of dependency syndrome on socio-demographic profile and pattern of substance use. Increasing age was significantly associated with increased likelihood of dependency syndrome in univariate model but in multivariate model age was not significantly associated with dependency syndrome. The effect of age was found non-significant after adjustment for the years since substance use began. This highlights that the longer duration of substance, the more likely it is to lead to dependency syndrome. Positive family history of substance usage doubles the likelihood of developing dependency syndrome compared to those individuals who did not have the history of substance use among parents or siblings which is consistent with previous evidence [12]. Previous evidence showed that increasing education decreases the likelihood of areca nut use and dependency, however we did not observe any association between education and dependency [9].

Smokers were significantly more likely to have dependency syndrome as compared to areca nut only and areca nut users with tobacco additives, this could be explained by the effect of nicotine in cigarette and has been well documented[13,14]. Our analysis illustrated that smokers were eight times more likely to develop the syndrome as compare to areca nut users only and two times more likely to as compared to areca nut users with tobacco additives. On the basis of our knowledge this is the first study in South Asian population which had direct comparison among the three groups of substance users. We had a reasonably larger sample size to estimate any difference between the groups.

### Limitations of this research

This study has several limitations which need to be mentioned. As the participants were selected by convenient sampling and from one region of this city, so given the sample limitations one can not draw conclusions about the population level estimates from this study. We used only male individuals; therefore, it is difficult to assume that the frequency of dependency syndrome would also be similar among the female population in this country. Most of the individuals included in this study were from average or below average socio-economic class, so the results are not generalisable to all the social classes of this country. We used a cross-sectional design so our study was unable to explore any time trends in use and the development of dependency syndrome among areca nut users.

### Public health implications

Pattern of areca nut use varies among different regions as well as groups of population. Its use may lead to oral diseases and malignancy, adverse pregnancy outcome and systemic diseases as well [12]. Areca nut use poses significant and avoidable morbidity and mortality. The present findings suggest that areca nut with and without tobacco additives is associated with dependence. Health education and awareness are perhaps the most crucial interventions required to be delivered so that the adverse effects of the substance can be appreciated by the community. Addressing a culturally sanctioned substance in terms of addiction and harm may meet with considerable resistance, especially if attention resulted in compromised supply or increased cost [12]. These interventions need to be focused on all age groups, in particular early age school children to avoid the future morbidity and mortality associated with this substance.

## Conclusions

Areca nut with and without tobacco additives is significantly associated with the dependency syndrome. In comparison to areca nut only users the smokers are eight times more likely to develop dependence while areca nut users with tobacco additives are also significantly more likely to have the dependence.

## Competing interests

The authors declare that they have no competing interests.

## Authors' contributions

SSM and KS conceived the idea and design of this study. MIA and KS were involved in the drafting of questionnaires and collection of data. PV and MIA analysed the data and written the initial draft of this manuscript. All authors were involved in the interpretation of data and the critical revision of the manuscript for important intellectual content. All authors approved the final draft for publication.
